# A new, easy-to-make pectin-honey hydrogel enhances wound healing in rats

**DOI:** 10.1186/s12906-017-1769-1

**Published:** 2017-05-16

**Authors:** Gessica Giusto, Cristina Vercelli, Francesco Comino, Vittorio Caramello, Massimiliano Tursi, Marco Gandini

**Affiliations:** Department of Veterinary Sciences, University of Torino, Largo P. Braccini 2-5, Grugliasco, (TO) Italy

**Keywords:** Hydrogel, Honey, Pectin, Rat, Wound

## Abstract

**Background:**

Honey, alone or in combination, has been used for wound healing since ancient times and has reemerged as a topic of interest in the last decade. Pectin has recently been investigated for its use in various biomedical applications such as drug delivery, skin protection, and scaffolding for cells. The aim of the present study was to develop and evaluate a pectin-honey hydrogel (PHH) as a wound healing membrane and to compare this dressing to liquid honey.

**Methods:**

Thirty-six adult male Sprague-Dawley rats were anesthetized and a 2 × 2 cm excisional wound was created on the dorsum. Animals were randomly assigned to four groups (PHH, LH, Pec, and C): in the PHH group, the pectin-honey hydrogel was applied under a bandage on the wound; in the LH group, liquid Manuka honey was applied; in the Pec group, pectin hydrogel was applied (Pec); and in the C group, only bandage was applied to the wound. Images of the wound were taken at defined time points, and the wound area reduction rate was calculated and compared between groups.

**Results:**

The wound area reduction rate was faster in the PHH, LH, and Pec groups compared to the control group and was significantly faster in the PHH group. Surprisingly, the Pec group exhibited faster wound healing than the LH group, but this effect was not statistically significant.

**Conclusion:**

This is the first study using pectin in combination with honey to produce biomedical hydrogels for wound treatment. The results indicate that the use of PHH is effective for promoting and accelerating wound healing.

## Background

Wound healing is a complex process that involves a plethora of factors that significantly influence the reestablishment of the skin barrier. Currently, several compounds are used to positively influence the wound healing process including honey [1]. The use of honey in wound healing, alone or in combination with other compounds, is ancient and has become a topic of interest in several investigations in the last decade [2, 3]. Honey contains high levels of glycine, methionine, arginine, and proline, which are all necessary for collagen formation and fibroblast deposition, the essential factors needed for healing [4]. Manuka honey has been demonstrated to have positive effects on wound heling [5].

During the wound healing process, the epithelium cells must be allowed to migrate, but this is only possible if the environment is moist. Hence, some of the most widespread dressing methods involve the use of hydrogels. Hydrogels aid in maintaining a moist environment, therefore facilitating wound healing by preventing dehydration, necrosis, and apoptosis [6]. Hydrogels have high water content and can absorb a large amount of body fluid, contributing to the maintenance of a moist environment and encouraging granulation tissue formation. Moreover, the tridimensional structure of hydrogels works as a scaffold, permitting cell adhesion, proliferation, and neoangiogenesis [6].

Pectin has recently been investigated for use in various biomedical applications including drug delivery, skin protection, and scaffolding [7]. Pectin is a heterosaccharide found in the terrestrial plant cell wall. It is a polyuronate, and when subjected to calcium-induced gelation, forms an egg box-like structure that enables cells inside the gel [8]. However, it is typically used in conjunction with other polymers because of its poor intrinsic mechanical properties [8]. Pectin is inexpensive, can be extracted from renewable sources, is not cytotoxic, acts as a gelating agent, and is suitable for many biomedical applications [9].

Based on the positive wound-healing properties of both honey and pectin, we hypothesized that a novel, hybrid wound dressing could be used to further enhance the regenerative process. The aim of the present study was to develop and evaluate a pectin-honey hydrogel (PHH) wound membrane and compare its effectiveness to pectin hydrogel (PH) and liquid honey.

## Methods

Honey (Medihoney 440) was purchased from Manuka Health (66 Weona Court, Te Awamutu 3800, New Zealand) and citrus pectin was purchased from Ardets.r.l. (Villanova Mondovì, Cuneo, Italy).

### Preparation of pectin-honey hydrogels (PHH) and pectin hydrogels (pec)

The preparation method used has been previously described, with some modifications [10, 11]. Briefly, the pectin-honey hydrogels were prepared starting from a solution (1:1 *v*/v) of liquid honey (Manuka Health, New Zealand) and sterile deionized water. The same volume of pectin powder^1^ was then added little by little with continuous stirring until the mixture was homogeneous. The resulting foam was spread onto 2 mm-thick films and hot-air-dried at 40 ± 0.5 °C and it was cut into squares of 5 × 5 cm and further conditioned in an air drier at 25 ± 1 °1 dr 5 days. The films were then collected and hand packed in polyethylene under vacuum. The pectin hydrogel (Pec) was made using the same method but substituting honey with the same volume of deionized water.

All films were sterilized by gamma-irradiation at 25 KGray (Sterigenics International LLTC, Bologna, Italy) [11, 12].

### Animals

All procedures were approved by the Bioethical Committee of the University of Turin and by the Italian Ministry of Health (In Italy the approval code it started in 2015).

Thirty-six adult male Sprague Dawley rats, weighing 225–250 g, were purchased from Charles-Rivers (Italy).

All rats were housed in single cages for 7 days prior to the beginning of the experiment. They were fed commercial food, and water was given ad libitum. The room temperature was set to 23 °C for the duration of the experiment, and cages were cleaned daily.

### Experimental wound model and wounding procedure

A full thickness excisional model was used to create the wounds [4]. Anesthesia was administered intramuscularly using 5 mg/kg of xylazine^2^ and 50 mg/kg of tiletamine and zolazepam.^3^ Animals were anesthetized for approximately 1 h. Under anesthesia, the dorsal hair was shaved^4^ and skin was cleaned using 3 steps with an iodiopovidone-clorexydine scrub. Using a dermatological pencil,^5^ a 2 × 2 cm square was drawn on the back skin, distally to the shoulder blades, and the skin was cut using a scalpel and scissors. This location was chosen because this area is seldom deformed by animal movements, preventing auto traumatism. Immediately after the surgery, all animals were dressed using a bandage without glue covered by a Vetrap.^6^

Animals were randomly divided into four groups of 9 animals each, using a calculator (https://www.random.org/integers/):

- Group C: negative control group. No treatment was applied.
- Group LH: liquid Manuka honey (from the same lot used for PHH production) was applied to the wounds before bandaging.
- Group Pec: animals treated with a pectin hydrogel under the dressing.
- Group PHH: animals treated with PHH under the dressing.

### Determination of the wound healing rate

On days 0, 2, 4, 6, 8, 11, 13, 15, 18, 21, and 23 after surgery the bandages were removed and digital pictures of the wounds were taken. Then, a new bandage with (groups Pec, PHH, and LH) or without (group C) treatment was applied. The animals were sedated with xylazine^2^ in order to perform the procedure. Photographs were taken in standardized conditions. Rats were gently held in the same position by an operator and a distance of 10 cm between the camera and dorsum of the rat was maintained. The wound surface area was then measured using Image J software.^7^ The comparison between the area at day 0 and at the time-set days was used to calculate the ratio of the wound reduction using the following formula:

$$ Wound\  area\  reduction\  rate=\left(\frac{ A t}{A0}\right)*100 $$

Where A_0_ and A_t_ are the initial area and the wound area at time t, respectively [4, 13].

### Histological analysis

After euthanasia, the area around the scar or residual wound was harvested, fixed in 4% buffered formalin, dehydrated, and fixed in paraffin. Five-micron slices were then stained with hematoxylin and eosin and evaluated by a blinded pathologist.

#### Statistical analysis

Data were analyzed with the Shapiro test to evaluate their distribution, and statistical differences were measured using one-way ANOVA for parametric values. Statistical significance was defined as *p* < 0.05. All tests were run using commercial software.^8^ The results are expressed as mean values ± standard deviation (SD).

## Results

All data passed the Shapiro-Wilk test and were normally distributed.

### Wound area reduction rate

**Fig. 1:**
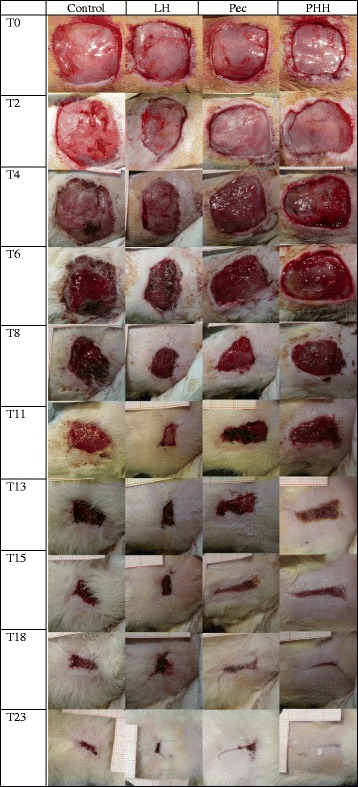
Picture of wound healing of different groups at each time point

The wound area reduction rates of the control and treatment groups are shown in Table 1 and Fig. 1. As shown, WARR was negative for all groups in the first 3 days than started to be positive. Total closure of the wound was completed for all groups but controls by day 23.

**Table 1:** Wound area reduction rate at each time point (±SD)

	Control	LH	Pec	PHH
Day 2	−4.44 ± 14.94	−2.95 ± 11.6	−8.37 ± 21.7	3.11 ± 24.59
Day 4	−11.88 ± 7.42	−11.06 ± 11.84	−9.25 ± 16.23	−13.52 ± 7.74
Day 6	0.42 ± 17.73	−1.04 ± 4.55	2.6 ± 3.01	−2.93 ± 2.48
Day 8	29.45 ± 19.26	37.35 ± 12.92	46.92 ± 12.98	30.3 ± 12.17
Day 11	55.33 ± 21.03	68.15 ± 13.19	64.58 ± 10.26	62.75 ± 7.69
Day 13	69.26 ± 8.71	72.55 ± 9.48	73.5 ± 5.95	81.81 ± 4.76
Day 15	73.93 ± 4.34	75.37 ± 12.01	78.88 ± 3.9	82.05 ± 3.81
Day 18	80.52 ± 4.71^a^	83.58 ± 5.06	88.56 ± 9.91	93.12 ± 3.93^a^
Day 21	83.39 ± 1.33^b^	86.96 ± 5.01^c^	89.8 ± 5.91	94.75 ± 3.79^b,c^
Day 23	88.22 ± 6.64^d^	94 ± 2.93	93.64 ± 3.79	99.17 ± 2.04^d^

^a^ *p* = 0.0182

^b^ *p* = 0.001

^c^ *p* = 0.0219

^d^ *p* = 0.0022

### Histological analysis

**Fig. 2:**
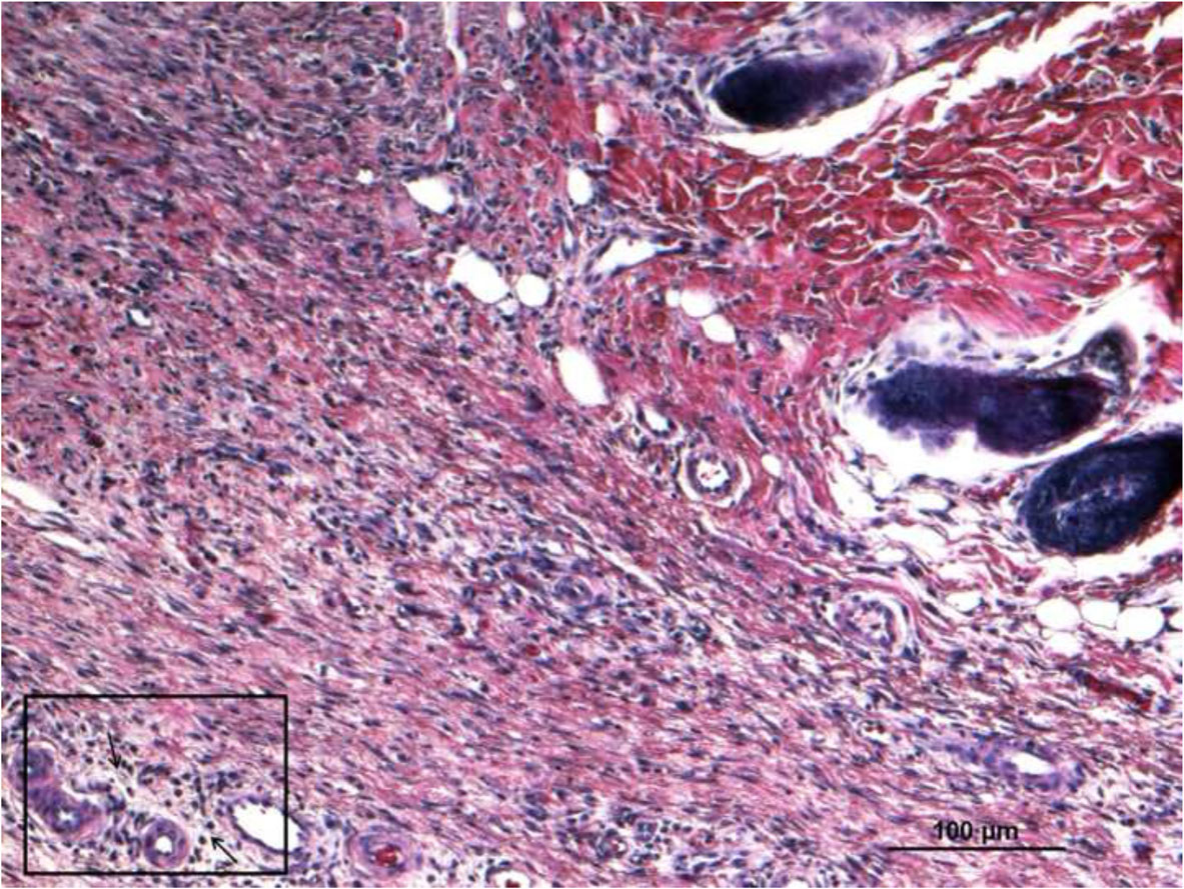
Histology image of a completed healed wound with organized mature fibrous tissue (*small arrows* in the box) and hair follicles (group PHH)

**Fig. 3:**
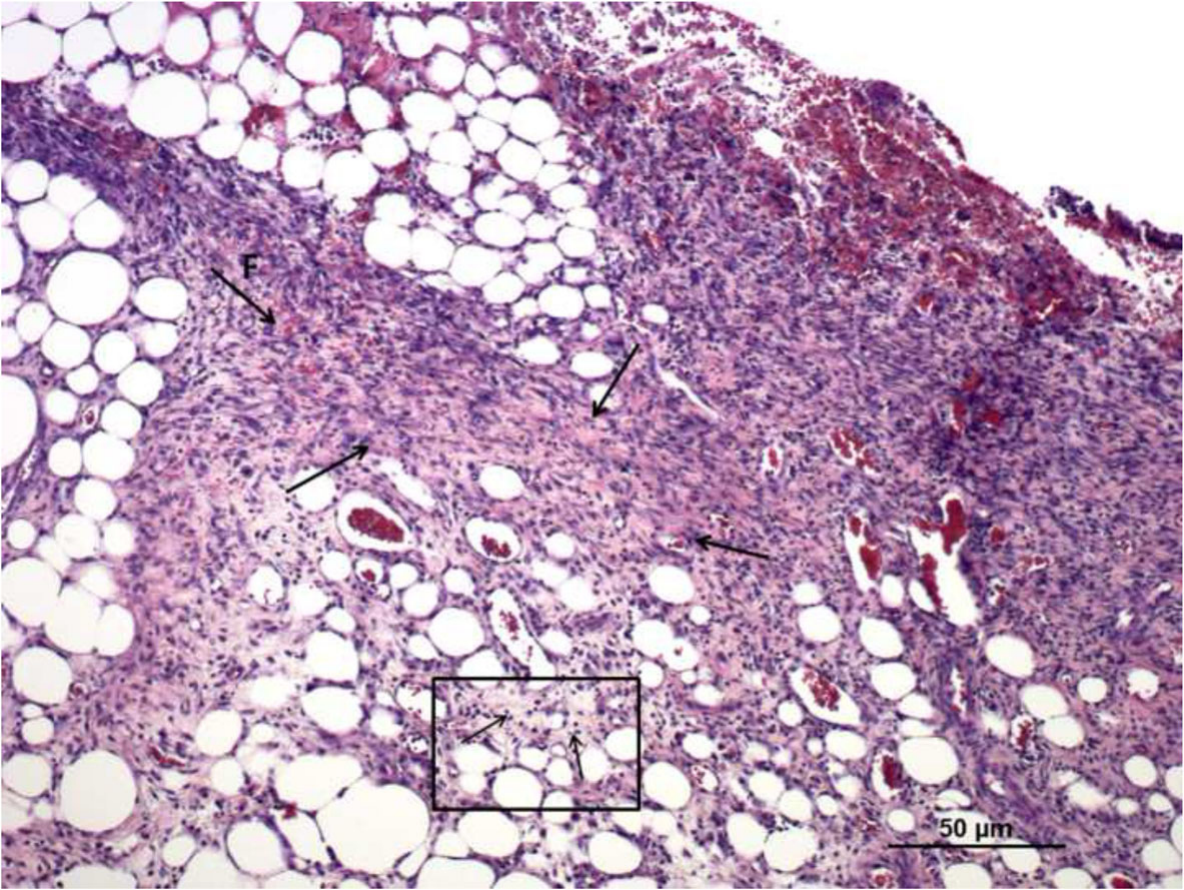
Histology image of the healed wound with severe dermal fibrosis (F and *large arrows*) and interstitial lymphocytic infiltration (*small arrows* in the box) (Control group)

**Fig. 4:**
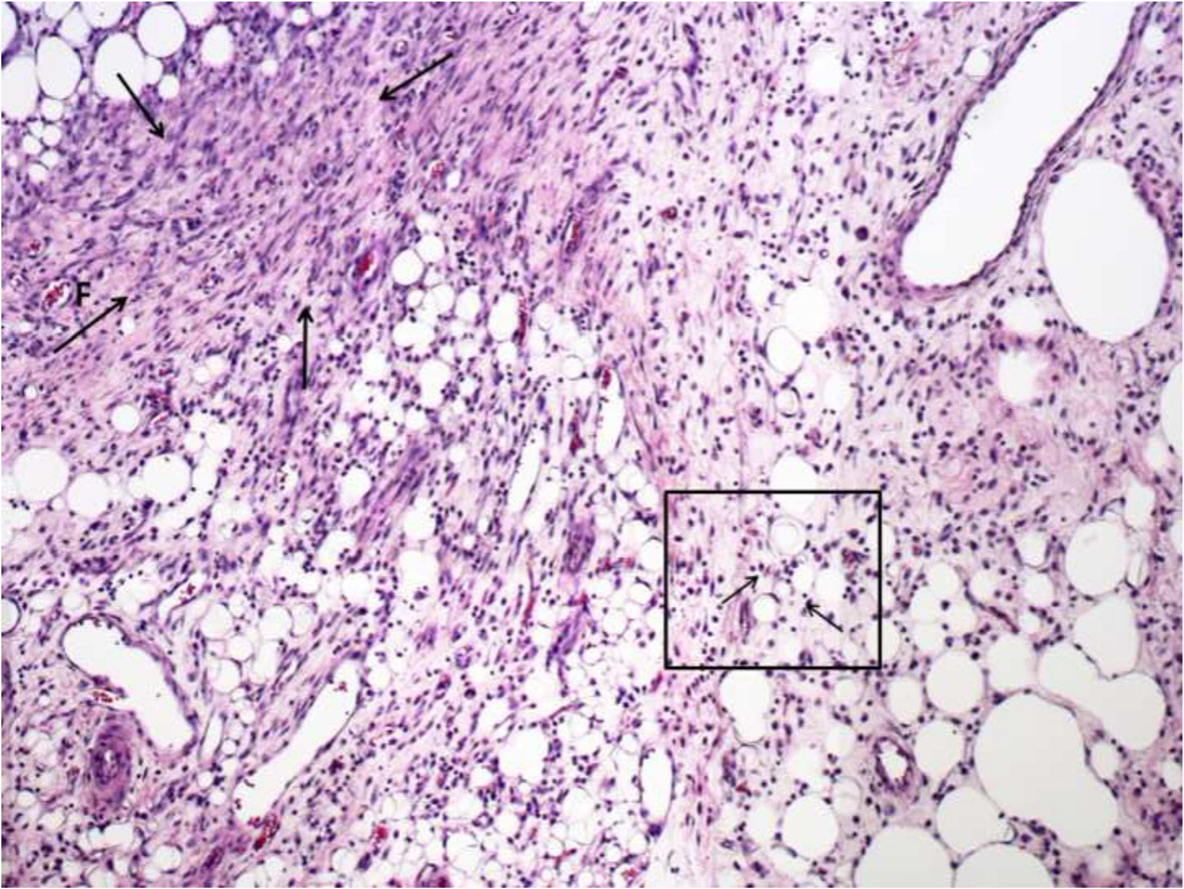
Histology image of the healed wound with moderate interstitial lymphocytic infiltration (*small arrows* in the box) and dermal fibrosis (group PHH)

**Fig. 5:**
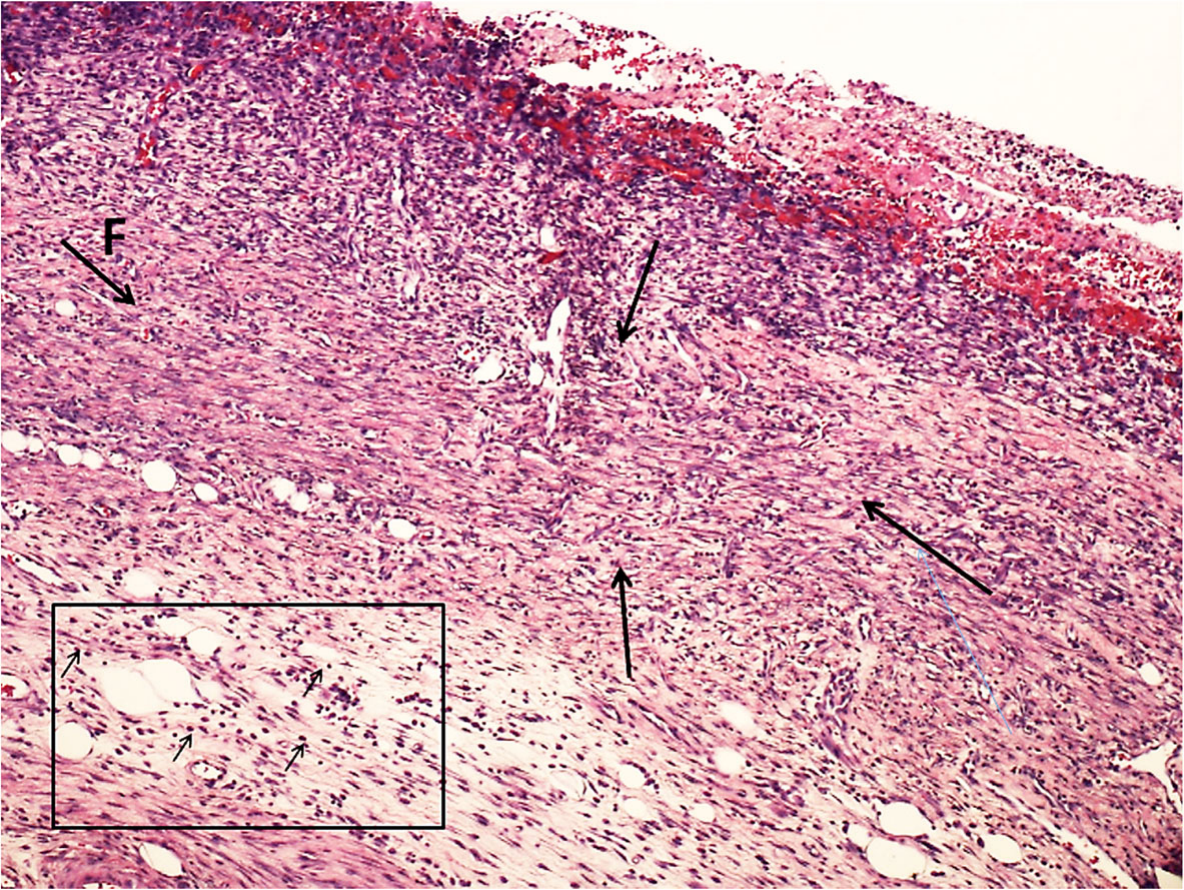
Histology image of the healed wound with severe interstitial lymphocytic infiltration (*small arrows* in the box) and dermal fibrosis (F and large arrows) (group LH)

On the 23rd day, the entire surface of the lesion treated with the dressing was covered with new epithelium. All the wounds treated with PHH and pectin dressing had well-developed dermis. Mature fibrous tissue proliferation was observed in the dermis. In the PHH and Pec groups, effective healing of the wounds was indicated by the presence of hair follicles and matured fibrous tissue (Fig. 2). In the control group, there was a significantly larger number of inflammatory cells compared with the treatment groups (PHH/Pec/LH) (Figs. 3, 4, and 5).

## Discussion

Incisional and excisional wounds are the two main models which allow for the determination of wound healing phases [4, 8]. Full thickness excisional wounds were used in this study to macroscopically evaluate the wound area reduction rate in animals treated with PHH, liquid honey, and pectin hydrogels. The results demonstrate that topical administration of pectin and pectin-honey hydrogels accelerates wound healing in rats. As reported in Table 1, we found that in the first 3 days there was an increase in the wound area. In our opinion, this was due to the dimensions of the wound that initially caused wound enlargement from the midline to the abaxial edges, caused by gravity forces. After six days the WARR started to be positive for all groups. Although not significantly, from this time on, the WARR was higher in all treated groups than in the control group. The difference become significant only from day 18. This could imply a positive effect of all treatments, but in particular of LH and PHH in the proliferative phase of the wound healing. Further, we found that the WARR was slower in the control group than what has been reported in previous studies [4, 13], and while the cause of this delay is unclear, could have contributed to the differences found.

The belief that keeping a wound dry promotes healing has been negated over the last several years [14]. A moist dressing provides a better environment for wound healing, which involves different steps such as cell migration, cell differentiation, angiogenesis, matrix formation, granulation tissue formation, and re-epithelialization. Epithelialization occurs faster in a wet environment, which can be created by an occlusive or semi-occlusive wound dressing [15]. The ideal dressing should be able to absorb the exudates on the wound surface [1]. For effective wound healing, this process should be promoted and not inhibited [14].

In a previous study, we demonstrated that a pectin-honey hydrogel has optimal characteristics for wound healing, in regards to the water vapor transmission rate (WVTR) and fluid uptake [10]. Honey, with its high concentration of sugar, is a hyperosmotic substance which has high hygroscopic capacity [16–18]. Honey is able to increase its weight under physiological conditions up to 150%, resulting in a substance that will likely be able to absorb excessive wound exudates [11]. Furthermore, hydrogels have been proven to have a good fluid absorbance, as a result of their hydrophilic nature, and this property is very important for quick absorption of exudates during the wound healing phases [1, 16]. Pectin can act as a scaffold for cell migration and differentiation [8], while honey acts as an anti-inflammatory, antibacterial, and stimulatory agent [4]. Acceleration of wound healing could be due to intrinsic characteristics of honey such as production of hydrogen peroxide and its nutritional, hydroscopic, antioxidant, and antibacterial properties, providing wounds a suitable healing environment [4]. Surprisingly, the pectin hydrogel performed better than bulk honey. This finding could be attributed to the natural properties of this substance, such as hydrophilicity, which create a barrier against bacteria. Pectin also becomes a binding agent for growth factors [9]. During pectin solubilization, the wound environment becomes acidic, which may help to control bacterial growth [9]. Another important advantage could be the direct and continuous contact of the hydrogel with the wound during the healing phase compared to bulk honey.

Wound contraction is an essential process in healing that leads to wound closure, and honey can increase contraction and enhance the deposition of fibroblasts and collagen, which are essential for healing [4]. It has been demonstrated that the greater the wound contraction, the lesser the scar deposition [19]. We believe that the increase in the wound reduction rate in the treated groups was caused by an increase in wound contraction caused by honey and the use of the scaffolding material pectin. The use of both materials allowed for the establishment of an ideal environment for healing, as demonstrated in previous studies [8, 9, 18, 20, 21]. The same factors may have caused the reduction in inflammation (compared with controls) found in the treatments groups.

The difference between the PHH and LH groups could be attributed to the sustained contact of honey with the wound as a result of the use of the pectin hydrogel and regenerative factors from the pectin itself.

Further studies on the effects of pectin hydrogel on wound healing are warranted to clarify this aspect.

The main advantages of PHH compared with other hydrogels or honey-based devices are that it is very inexpensive, easy to produce, and is easily applied to the wound. This should allow for the use of honey membrane wound dressings in economically disadvantaged regions. The most expensive material in the membrane composition is Manuka honey, responsible for its antimicrobial activity and for the improvement in the wound healing process [13, 22]. Several investigators worldwide are studying the different characteristics of honeys, and it will be possible to utilize less expensive honeys in the pectin-honey hydrogel in the future [18, 23–25].

## Conclusions

This is the first study that uses pectin in combination with honey to produce biomedical hydrogels for wound treatment. Our results clearly indicate the synergistic effect of materials used for preparation of the films, and each material contains a large number of healing-promoting activities that can be found separately in pharmaceutical products. These combined materials could be used in wound healing applications. Based on the results obtained in the present study, the use of PHH is effective for promoting and accelerating wound healing.

## Footnotes

1 ARDET s.r.l., Cuneo, Italy

2 Bayer Animal Health, Milano, Italy

3 Virbac, Milano, Italy DermaSciences, Princeton, NY

4 Reckitt Benckise, Milano, Italy

5 Conmed, Utica, NY, USA

6 3 M, Italy

7 National Institutes of Health, USA

8 GraphPad Software Inc., La Jolla, USA
